# Jujuboside A Exhibits an Antiepileptogenic Effect in the Rat Model via Protection against Traumatic Epilepsy-Induced Oxidative Stress and Inflammatory Responses

**DOI:** 10.1155/2022/7792791

**Published:** 2022-09-09

**Authors:** Wei Lu, Zhangze Wu, Chong Zhang, Tingting Gao, Xiaoyang Ling, Min Xu, Wenhua Wang, Xuegang Jin, Keran Li, Long Chen, Jinjuan Wang, Zhongyang Sun

**Affiliations:** ^1^Department of Neurosurgery, Kunshan Hospital of Traditional Chinese Medicine, Nanjing University of Chinese Medicine, Kunshan, China; ^2^Department of Neurosurgery, Air Force Hospital of Eastern Theater, Nanjing University of Chinese Medicine, Nanjing, China; ^3^The First Retired Cadres' Sanatorium of Jiangsu Military Region, Nanjing, China; ^4^Faculty of Life Science, School of Biomedical Science, University of Bristol, Bristol, UK; ^5^Department of Pharmacy, Nanjing First Hospital, Nanjing Medical University, Nanjing, China; ^6^Department of Orthopedics, Air Force Hospital of Eastern Theater, Nanjing University of Chinese Medicine, Nanjing, China; ^7^Department of Orthopedics, Air Force Hospital of Eastern Theater, Anhui Medical University, Nanjing, China; ^8^Department of Orthopedics, Affiliated Jinling Hospital, Medical School of Nanjing University, Nanjing, China

## Abstract

Traumatic brain injuries (TBI) are the greatest source of death in trauma, and post-traumatic epilepsy (PTE) is one of the common complications of TBI. Oxidative stress and inflammatory responses play an important role in the process of PTE. Many studies have shown that Jujuboside A has powerful antioxidant and anti-inflammatory properties. However, it is not known whether Jujuboside A has an anti-epileptic effect. The influences of Jujuboside A in the experimental FeCl_3_-induced model of PTE were tested by estimating the grade of seizures and performing behavioral tests. Following that, we detected oxidative stress indicators and inflammatory factors. Additionally, western blotting was used to test the protein levels of signaling molecules in MAPK pathways. In this study, Jujuboside A was found to have improved the recognition deficiency and epilepsy syndromes in the experimental rat model. Moreover, oxidative stress and inflammatory responses induced by FeCl_3_ injection were relieved by Jujuboside A. In addition, Jujuboside A was found to be capable of reducing the increased expression of p-P38 and p-ERK1/2 caused by iron ions. Collectively, our results demonstrated that Jujuboside A exhibits an antiepileptogenic effect by alleviating oxidative stress and inflammatory responses via the p38 and ERK1/2 pathways.

## 1. Introduction

Post-traumatic epilepsy (PTE) is a kind of acquired epilepsies caused by traumatic brain injury (TBI), which is one of the common complications of brain injury [1–3]. The data showed that approximately 10–25% of patients gradually develop epilepsy within 10 years after TBI [1–3]. PTE can cause a variety of pathological changes such as the abnormal release of neurotransmitters, sprouting of axons, neuronal death, oxidative stress, and inflammatory responses [4].

FeCl_3_ injection-induced epilepsy is a widely used and stable animal model of human PTE [5]. The iron injection in the cortex can initiate oxidative stress and inflammatory responses in the brain of rats, which will cause electro-behavioral seizure induction [6]. The mechanism of epilepsy, induced by the iron injection, is similar to the clinical PTE [6]. Therefore, the iron injection model is widely used for the investigation of human PTE and its pharmacology [7].

Although there are numerous mechanisms of PTE, increasing evidence showed that oxidative stress and inflammatory responses play an increasingly important role in the development of PTE [8–13]. A large number of reactive oxygen species (ROS) was produced in brain tissue after PTE. Excessive ROS can induce lipid peroxidation and inactivate antioxidant stress-related proteins, which will aggravate TBI injury [8]. Therefore, PTE can induce lipid peroxidation in the biofilm. Particularly, the levels of F_2_-isoprostanes, nicotinamide adenine dinucleotide phosphate (NADPH) oxidase activity, and malondialdehyde (MDA) are the most commonly used indicators of oxidative stress [9]. When ROS injury occurs, the body can spontaneously initiate a variety of defense mechanisms. Thus, the levels of antioxidants such as superoxide dismutase (SOD), catalase (CAT), and glutathione peroxidase (GSH-Px) are also usually regarded as indicators of oxidative stress injury [10]. Additionally, excessive production of ROS can stimulate tissues to produce and release a variety of inflammatory cytokines, such as interleukin 1 beta (IL-1*β*), interleukin 6 (IL-6), and tumor necrosis factor alpha (TNF-*α*) [11]. On top of that, the myeloperoxidase (MPO) activity is an indicator of neutrophil infiltration and inflammatory cytokine activation in the FeCl_3_ injection-induced epilepsy animal model [12, 13]. Moreover, when oxidative stress or inflammatory responses occurs, the mitogen-activated protein kinase (MAPK) pathways are activated. Specifically, several studies have described that the activation of p38 and ERK1/2 is involved in the brains of epileptic rats [14, 15]. It has also been reported that the iron injection model has been proven to activate the p38 and ERK1/2 pathways [6, 7, 16].

Jujuboside A (Figure S1) is the main active ingredient of semen Ziziphi Spinosae, the mature seed of Ziziphus Jujuba Mill var. Spinosa (Bunge) Hu ex. H. F. Chou (Rhamnaceae), which is widely used as a traditional Chinese herb. The pharmacological study of semen Ziziphi Spinosae showed that its effects included lowering blood pressure and blood lipid, antimyocardial ischemia, antiarrhythmia, antiaging, antiradiation, antihypoxia, antioxidation, antiinflammation, sedation, hypnosis, antianxiety, and anticonvulsion [17–22]. It has been reported that Jujuboside A attenuates heart failure-related injury through its antioxidant and anti-inflammatory properties [18]. However, there are only a few reports on whether Jujuboside A has an antiepileptic effect. In this study, we discovered that Jujuboside A could improve the recognition deficiency in an experimental rat model of FeCl_3_ injection-induced epilepsy. Additionally, oxidative stress and inflammatory responses induced by FeCl_3_ were relieved by Jujuboside A. Moreover, Jujuboside A treatment could inhibit P38 and ERK1/2 pathway activation induced by iron ions.

## 2. Materials and Methods

### 2.1. Materials

Jujuboside A (purity ≥99.5%) (cat. No. HY-N0659) (MedChemExpress, China); NADPH oxidase activity (cat. No. A012-1-3), F_2_-isoprostane (cat. No. A022-4-1), MDA (cat. No. A004-3-4), SOD (cat. No. A001-3-2), GSH-Px (cat. No. A005-1-2), and CAT (cat. No. A007-1-1) assay kits (Nanjing Jiancheng Biotechnology Institute, China); MPO (cat. No. H074), TNF-*α* (cat. No. H052), IL-6 (cat. No. H007), and IL-1*β* (cat. No. H002) ELISA kits (Nanjing Jiancheng Biotechnology Institute, China); SB203580 (P38 MAPK pathway specific inhibitor) (cat. No. HY-10256) and ravoxertinib (ERK pathway specific inhibitor) (cat. No. HY-15947) (MedChemExpress, China); anti-p-P38 (cat. No. ab4822) (1 : 1,000 dilution), anti-p-ERK1/2 (cat. No. ab223500) (1 : 400 dilution), anti-*β*-tubulin (cat. No. ab8227) (1 : 5,000 dilution), and horseradish peroxidase-labeled secondary antibody (cat. No. ab6721) (1 : 10,000 dilution) (Abcam, USA); a BCA kit (cat. No. R0054), RIPA buffer (cat. No. R0010), and loading buffer (cat. No. R0122) (Beyotime, China).

### 2.2. Animals and Ethics Statement

This study was performed with male Sprague-Dawley rats aged 6 weeks, weighing 150–200 g. All rats were obtained from the Nanjing University of Chinese Medicine Laboratory Animal Center. All the rats were housed in a plexiglass cage under standard environmental conditions of temperature (25 ± 2°C) and humidity (55 ± 2%) with a 12-hour light/dark cycle and fed with standard food pellets with access to sterile water *ad libitum*. Animal experiments were conducted during the daytime. Surgical procedures were performed under mild anesthesia produced with ketamine (80 mg/kg) and xylazine (10 mg/kg). The rats were killed by decapitation under isoflurane anesthesia, and the hippocampal sections were dissected and stored at −80°C until use. All experimental procedures were approved by the Ethics Committee for Animal Use of the Nanjing University of Chinese Medicine (Reference number: 20190305014).

### 2.3. Surgical Procedure and Recordings

The surgical procedures for the preparation of the rat model were described in previous studies [23]. The stereotactic method was used to mark the location of the hole. A hole with a diameter of 0.5 mm was drilled on the surface of the rat skull with a drill. Electrodes were placed and an intracortical injection was performed. Each electrode was wired to an adapter. Then, an acrylic adhesive was used to attach the connector to the skull surface. Seizure was induced by injection of FeCl_3_ into the somatosensory area of the brain. A volume of 5 *μ*l, 100 mM FeCl_3,_ or normal saline was injected through the hole in the somatosensory region of the cortex for 5 mins at a rate of 1 *μ*l/min. The holes were closed with bone wax after the injection. The electrodes were stereotaxically implanted into the cortex [23]. After the rats recovered from surgery, Somnologica studio software (Embla, USA) was used to record the continuous synchronous video and electroencephalogram (EEG). The bilateral cortical electrical cortex was recorded from the ipsilateral (FeCl_3_ injection side) and the contralateral cortex. The EEG difference was recorded to reduce EEG artifacts. Each rat received 8 hours of video/EEG monitoring within 1 week for 20 weeks. In this study, the modified form of the Racine kindling behavior score was used to classify the grade of epileptogenesis [24].  Table S1 lists the criteria used for classifying different seizure grades [23, 24].

### 2.4. Experimental Groups and Drug Treatment

The animals were randomly divided into the following three groups: (1) the control group (sham operation), (2) the epileptic group (FeCl_3_ injection), and (3) the Jujuboside A group (FeCl_3_ injection + 0.02 mg/kg/d). Jujuboside A was dissolved in saline solution. Because Jujuboside A is a macromolecular polyphenol compound, it has been predicted that only a limited amount of it would be able to penetrate the blood brain barrier. In order to increase the drug content in the central nervous system, we chose intracerebroventricular (ICV) microinjection as the route of administration. This method is widely used in clinics [17, 18]. The Jujuboside A group rats were given 0.02 mg/kg of Jujuboside A for five consecutive days, starting one day after the completion of surgical procedures, whereas the control group and the epileptic group rats were given equal volumes of saline. In most studies, ICV microinjection of 0.02 mg/kg/d of Jujuboside A has been used as the effective route and concentration of administration for experiments [17, 18]. Therefore, we chose ICV microinjection of 0.02 mg/kg/d of Jujuboside A in this study to further explore the influences of Jujuboside A in PTE. Notably, ICV microinjection of 0.02 mg/kg of Jujuboside A for five consecutive days had no effects on the healthy rats (Figure S2).

### 2.5. Morris Water Maze Task

The parameters of the maze were 168 cm in diameter and 50 cm in depth. There were extra maze clues of different colors and shapes in the maze. In the center of each quadrant, there was a black circular platform with a diameter of 15 cm, which was 2 cm underwater. This platform was for the rats to escape by swimming. The swimming ability of the rats was screened by recording the latency to the visible platform. The visual cues were used to train the rats to exit the water tank and enter the platform. Each rat was placed in one of four randomly selected entry points in the water maze, once in each block. Each rat was tested eight times a day for four consecutive days, and the latency of each test was recorded. The experimental steps of the Morris water maze task referred to a previously described study [23].

### 2.6. Elevated plus Maze Test

The maze consisted of two open arms and two closed arms, containing a square platform in the central intersection, which was 25 cm above the ground. Each rat was placed on one end of the open arm with its back facing the central platform of the maze. In the first trial, the time from the open arm to the complete the entry of the limbs into the closed arm was recorded as the initial transfer latency. After recording the time, the rats were allowed to explore the maze for 10 s. Twenty-four hours later, the retention transfer latency test was performed in the same way as in the acquisition trial. The experimental steps of the elevated plus maze test referred to previously described studies [25, 26].

### 2.7. Passive Avoidance Task

The device composes of two independent chambers, one is a bright chamber and the other is a dark chamber. Both chambers are connected by a guillotine door. At the beginning of the experiment, a rat was placed in the bright chamber. After 60 s, the guillotine doors were opened and the initial latency of the rats entering the chamber was recorded. When the rats entered the dark chamber, the doors of the guillotine were closed immediately and the grid plate was triggered to conduct an electric foot shock (75 V, 0.2 mA, 50 Hz) for 3 s. Five seconds later, the rats were taken out of the dark chamber and returned to the cage. Twenty-four hours later, the retention latency was measured in the same way as the acquisition test, but no foot shock was given. The experimental steps of the passive avoidance task referred to previously described studies [25, 27].

### 2.8. Measurement of Oxidative Stress Indicators and Inflammatory Factors

According to the manufacturer's instructions, NADPH oxidase activity, F_2_-isoprostane, MDA, SOD, GSH-Px, and CAT assay kits were used to assess the concentration of these oxidative stress indicators. Moreover, MPO, TNF-*α*, IL-6, and IL-1*β* ELISA kits were used to assess the levels of these inflammatory factors [15].

### 2.9. Western Blotting

A BCA kit was used to examine the protein concentration of the hippocampus tissue lysis and subsequent homogenization. Loading buffer was added to the protein samples and boiled for preservation. Then, the proteins with different molecular weights were separated by SDS/PAGE. Finally, the separated proteins were transferred to the nitrocellulose membrane. The nitrocellulose membrane was incubated overnight with a suitable concentration of a primary antibody at 4°C. The nitrocellulose membrane was incubated with the horseradish peroxidase-labeled secondary antibody at room temperature for 1 h and detected by using Tanon imaging software (Tanon, China) [15].

### 2.10. Statistical Analysis

All data were expressed as the means ± SEs. One-way analysis of variance (ANOVA) with the Bonferroni *post hoc* test was used to determine statistical differences in the grade of seizure and the mean latency to find a platform among groups. The ANOVA with the Tukey *post hoc* test was used to determine statistical differences of other data. All the analyses were performed with SPSS version 20.0 (SPSS, USA) and OriginPro version 2017 (OriginLab, USA). *P* < 0.05 was considered statistically significant.

## 3. Results

### 3.1. Jujuboside A Mitigates the Progression of Seizures in Epileptic Rats

**Figure 1:**
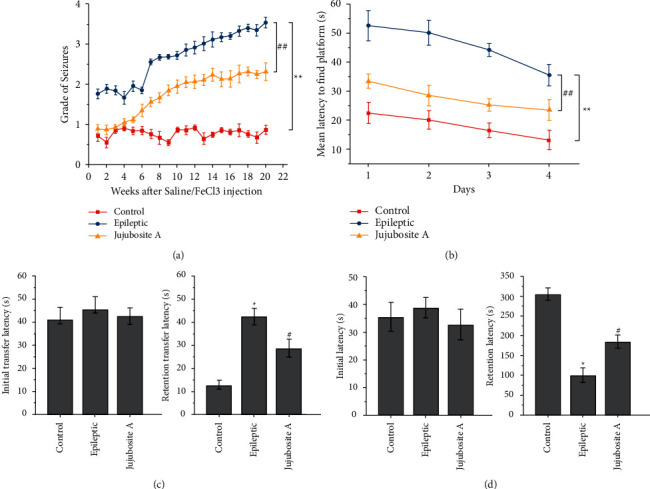
Jujuboside A ameliorates the progression of seizures and behavioral deficits in an experimental model of epilepsy. (a) The effects of Jujuboside A on the development of epileptiform activity in epileptic rats by the seizure grade. (b) The effects of Jujuboside A on the spatial learning and memory of rats in different groups using the Morris water maze task. (c) The effects of Jujuboside A on the memory of rats by the elevated plus maze test. (d) The effects of Jujuboside A on the ability of rats to retain and recall information by the passive avoidance task. ^*∗*^*P* < 0.05 and ^*∗∗*^*P* < 0.01 compared with the control group; ^#^*P* < 0.05 and ^##^*P* < 0.01 compared with the epileptic group; *n* = 8 per group. Jujuboside A ameliorates oxidative stress and inflammatory response in epileptic rats.

The development of epileptiform activity was evaluated by the seizure grade (Table S1), which was classified by the modified form of the Racine kindling behavior score [24]. In the Epileptic group, grade III and IV seizures occurred 12 weeks after FeCl_3_ injection. However, no grade III or IV seizure was detected in the control group and the Jujuboside A group (Figure 1(a)). Moreover, electrophysiological observations clearly indicated that Jujuboside A reduced the progression of seizures (Figure 1(a), *P* < 0.05).

### 3.2. Jujuboside A Alleviates Behavioral Deficits in an Experimental Model of Epilepsy

The Morris water maze task was used to assess the spatial learning and memory of rats in different groups [19]. The mean latency resulted in our conclusion that the platform was regarded as an indicator of spatial learning and memory. Rats in the epileptic group exhibited longer mean latency to find the platform compared with rats in the control group, which was reduced by Jujuboside A treatment (Figure 1(b), *P* < 0.05).

An elevated plus maze test was conducted to evaluate the memory of rodents [25, 26]. If the rats had previously experienced the test, the transfer latency would be shortened. We observed that there was no difference in the initial transfer latency from the open arm to the closed arm among each group (Figure 1(c)). However, FeCl_3_ injection induced an increase in the retention transfer latency. The rats were tested 24 h after initial transfer latency compared with the control group (Figure 1(c), *P* < 0.05). Jujuboside A treatment reduced the retention transfer latency compared with the epileptic group (Figure 1(c), *P* < 0.05).

The passive avoidance task has been employed to examine the ability of rats to retain and recall information [25, 27]. In the test, if the retention latency of the rats moving from the bright chamber to the dark chamber was prolonged, it indicated that the rats had gained the ability of learning and memory. The results showed that there was no difference in initial latency amongst the different groups (Figure 1(d)); whereas, FeCl_3_ injection decreased the retention latency in the passive avoidance paradigm compared with the control group (Figure 1(d), *P* < 0.05). When the rats were treated with Jujuboside A along with FeCl_3_ injection, it increased the retention latency compared with the epileptic group (Figure 1(d), *P* < 0.05).

**Figure 2:**
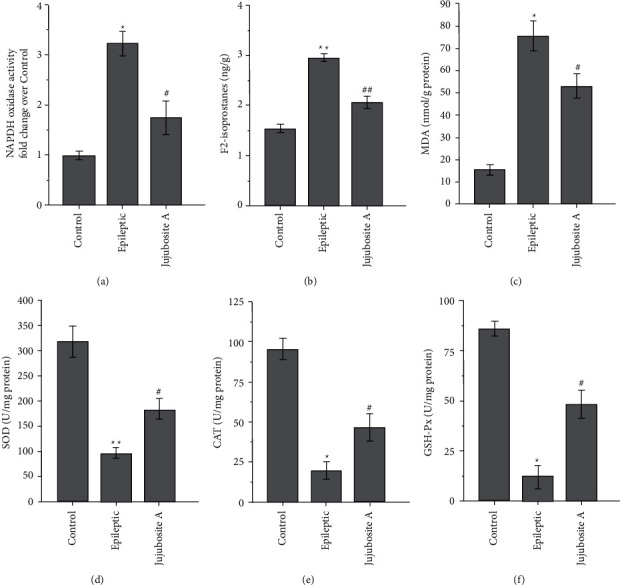
Jujuboside A reduces the levels of oxidative stress indicators and enhances the activities of antioxidant enzymes in epileptic rats. The activity of NADPH oxidase, (a) and the levels of F_2_-isoprostanes (b) and MDA (c) were higher in the epileptic group, while the activity of SOD (d) CAT (e) and GSH-Px (f) were lower in the Jujuboside A group. ^*∗*^*P* < 0.05 and ^*∗∗*^*P* < 0.01 compared with the control group; ^#^*P* < 0.05 and ^##^*P* < 0.01 compared with the epileptic group; *n* = 8 per group.

The indicators of oxidative stress (NADPH oxidase activity, F_2_-isoprostane levels, and MDA levels) were higher in the epileptic group than those in the control group. Moreover, the indicators of oxidative stress were lower in the Jujuboside A group than those in the epileptic group (Figures 2(a) and 2(c), *P* < 0.05). The activities of antioxidant enzymes (SOD, CAT, and GSH-Px) were lower in the epileptic group than those in the control group. Additionally, the activities of antioxidant enzymes were higher in the Jujuboside A group than those in the epileptic group (Figures 2(d)–2(f), *P* < 0.05).

**Figure 3:**
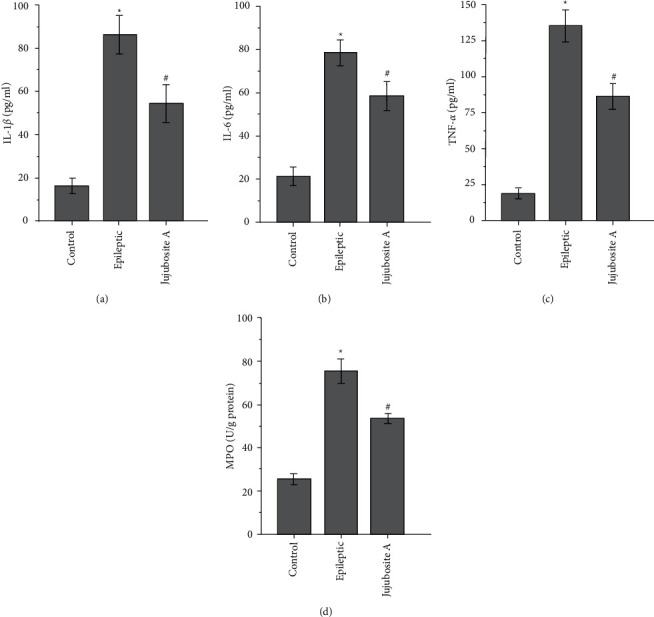
Jujuboside A relieves the inflammatory response in an experimental model of epilepsy. The levels of IL-1*β* (a) IL-6 (b) and TNF-*α* (c) and the activity of MPO (d) were higher after FeCl_3_ injection, but Jujuboside A alleviated these injection-induced increases in inflammatory indicators. ^*∗*^*P* < 0.05 compared with the control group; ^#^*P* < 0.05 compared with the epileptic group; *n* = 8 per group. Jujuboside A suppresses the activation of the p38 and ERK1/2 pathways induced by FeCl_3_ injection.

The inflammatory indicators (the levels of IL-1*β*, TNF-*α*, and IL-6, and the activity of MPO) were higher in the epileptic group than those in the control group (Figures 3(a) and 3(d), *P* < 0.05). In addition, treatment with Jujuboside A ameliorated an FeCl_3_ injection-induced increase in inflammatory indicators (Figures 3(a)–3(d), *P* < 0.05).

**Figure 4:**
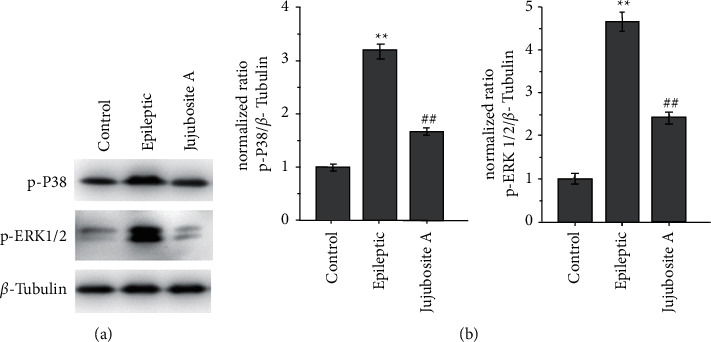
Jujuboside A suppresses the activation of the p38 and ERK1/2 pathways induced by FeCl3 injection. (a) Representative immunoblots of p-P38, p-ERK1/2, and *β*-tubulin in different groups (the uncropped western blot scans are shown Figure S4). The total amount of protein loaded per lane was 40 *μ*g. Detection of *β*-tubulin on the same blots was used to verify equal loading among the various lanes. (b) A bar graph illustrating the average relative expression of p-P38 and p-ERK1/2 in each group. The protein levels were quantified by camera-based detection of emitted chemiluminescence. ^*∗∗*^*P* < 0.01 compared with the control group; ^##^*P* < 0.01 compared with the epileptic group; *n* = 4 per group.

As shown in Figure 4, FeCl_3_ injection alone increased the expression of phosphorylated p38 and phosphorylated ERK1/2, while Jujuboside A treatment attenuated the FeCl_3_ injection-induced upregulation of phosphorylated p38 and phosphorylated ERK1/2 (Figures 4(a)–4(b), *P* < 0.05).

## 4. Discussion

In this study, we initially demonstrated that Jujuboside A mitigates the progression of seizures and alleviated behavioral deficits in epileptic rats. Furthermore, we found that Jujuboside A could ameliorate oxidative stress and inflammatory responses induced by FeCl_3_ injection. In addition, our findings suggested that Jujuboside A suppressed the activation of the p38 and ERK1/2 pathways in an experimental model of epilepsy, suggesting that the antiepileptogenic effect of Jujuboside A might be partly mediated by suppressing the activation of p38 and ERK1/2 pathways.

Several studies have described that PTE, a kind of acquired epilepsies caused by TBI, is an important clinical problem that should not be ignored [1–3]. Massive research indicated that there were different methods to treat PTE, mainly including drug therapy and nondrug therapy. It has been reported that rapamycin, carbamazepine, phenytoin sodium, sedative-hypnotic, levetiracetam, atorvastatin, losartan, curcumin, lipoic acid, and some other agents can exhibit the antiepileptogenic effect. In addition, gene therapy, stem cell therapy, and deep brain stimulation have been shown to inhibit the onset and progression of seizures [28–32]. However, the antiepileptic drugs (AEDs) are effective in suppressing epileptogenesis only when treatment is continued, and blood concentration remains sufficiently high. Moreover, prolonged treatment with AEDs produces unwanted side effects, such as chronic cognitive, memory, and behavior changes, severe hepatotoxicity and neurotoxicity, and teratogenic effects and withdrawal reactions [33, 34]. Additionally, the nondrug abovementioned therapy has shown some benefits in the laboratory, none of them have been established to have any clinical benefits [35, 36]. Hence, there is a growing interest to discover novel substances for alternative antiepileptogenic treatments.

Jujuboside A is one of the active ingredients of spine date seed, a Chinese herb used in the treatment of insomnia and anxiety [17]. In this study, we demonstrated that Jujuboside A inhibited the onset and progression of seizures and alleviated behavioral deficits in epileptic rats. Other studies have also shown that Jujuboside A could inhibit excessive brain activity and improve cognition [21]. Tabassum et al. have shown that Jujuboside A alleviated the disturbance of hippocampal neuronal excitability and memory impairment caused by sleep loss [18]. Zhang et al. reported that Jujuboside A ameliorated the cognitive deficiency in Alzheimer's disease [17].

Oxidative stress and inflammatory responses are considered to play central roles in the process of PTE [8–13]. Our findings suggested that Jujuboside A could ameliorate oxidative stress and systemic inflammatory responses in the FeCl_3_ injection-induced epilepsy rat model. Meanwhile, it was previously reported that Jujuboside A could notably reduce the myocardial damage caused by isoproterenol via anti-inflammatory and antioxidant effects [22]. Others demonstrated that Jujuboside A could protect neurons by reducing the activities of acetylcholinesterase and nitric oxide synthase via anti-oxidative and anti-inflammatory effects in dementia animals [21]. These findings suggested that the antioxidative and anti-inflammatory activities of Jujuboside A might be important in epileptic rats.

The MAPK pathway, such as p38 and ERK1/2 pathways, could be activated by oxidative stress or inflammatory response [14, 15]. Specifically, several studies have described that the activation of p38 and ERK1/2 was involved in the brain of epileptic rats. It has also been reported that iron injection could activate the p38 and ERK1/2 pathways [6, 7, 16]. In this study, we found that oxidative stress and inflammation were alleviated by blocking these two signaling pathways with their specific inhibitors (P38 pathway: SB203580 and ERK pathway: ravoxertinib) (Figure S3). Moreover, we demonstrated that Jujuboside A treatment partially inhibited the activation of p38 and ERK1/2 pathways induced by the FeCl_3_ injection, suggesting that inactivation of these signaling pathways might mediate some of the antiepileptogenic effects of Jujuboside A in this model. These observations are consistent with the findings of previous studies. Wan et al. showed that Jujuboside A inhibited norepinephrine-induced cardiomyocytes apoptosis by modulating p38 and ERK1/2 signaling pathways [20]. In another study, Zhang et al. showed that Jujuboside A ameliorated cognitive deficiency in Alzheimer's disease through mediating p38 and ERK1/2 signaling pathways [17].

There were some limitations to our study. We observed the involvement of the p38 and ERK1/2 pathways in the antiepileptogenic effect of Jujuboside A in the rat model. However, the specific effects of these pathways in FeCl_3_ injection-induced epilepsy remain to be investigated.

## 5. Conclusions

Our study demonstrated that Jujuboside A inhibits the onset and progression of seizures and alleviated behavioral deficits in epileptic rats. Moreover, Jujuboside A could ameliorate oxidative stress and inflammatory responses induced by FeCl_3_ injection via the p38 and ERK1/2 pathways. Jujuboside A may be a promising drug for PTE.

## Data Availability

We provided a portion of the raw data in the Supplementary Material. If necessary, we shall provide all the original data.

## Supplementary Materials

Supplementary Materials We provided Figures S1–S4 and Table S1 in the supplementary material for comprehensive analysis. Part of the raw data (analyzed by OriginPro) has also been provided in the.
