# Supplementary material for: Jujuboside A Exhibits an Antiepileptogenic Effect in the Rat Model via Protection against Traumatic Epilepsy-Induced Oxidative Stress and Inflammatory Responses
Source: Evid Based Complement Alternat Med. 2022 Sep 9;2022:7792791. doi: 10.1155/2022/7792791 (PMC9481365; doi:10.1155/2022/7792791)
Supplement: Supplementary Materials — We provided Figures S1–S4 and Table S1 in the supplementary material for comprehensive analysis. Part of the raw data (analyzed by OriginPro) has also been provided in the. [file 7792791.f1.zip › 7792791.f1/raw data.pdf]

figure 1-a

1	0.72	0.14	1.76	0.12	0.89	0.11
2	0.55	0.13	1.89	0.111	0.87	0.12
3	0.86	0.08	1.85	0.11	0.92	0.08
4	0.91	0.07	1.67	0.16	1.05	0.09
5	0.84	0.09	1.96	0.13	1.12	0.07
6	0.85	0.1	1.85	0.08	1.35	0.16
7	0.76	0.11	2.56	0.07	1.56	0.11
8	0.67	0.16	2.68	0.07	1.67	0.12
9	0.55	0.07	2.69	0.06	1.85	0.13
10	0.87	0.06	2.72	0.1	1.95	0.16
11	0.86	0.11	2.86	0.15	2.05	0.13
12	0.92	0.07	2.92	0.16	2.07	0.17
13	0.65	0.14	3.02	0.17	2.11	0.14
14	0.75	0.07	3.12	0.19	2.24	0.16
15	0.86	0.06	3.18	0.11	2.13	0.19
16	0.81	0.07	3.21	0.08	2.14	0.21
17	0.86	0.16	3.33	0.13	2.26	0.22
18	0.76	0.08	3.41	0.09	2.32	0.11
19	0.68	0.14	3.35	0.14	2.25	0.1
20	0.87	0.11	3.54	0.13	2.32	0.22

figure 1-b

1	22.53	3.65	52.62	5.21	33.5	2.36
2	20.12	3.23	50.21	4.25	28.56	3.54
3	16.56	2.56	44.23	2.21	25.3	2.12
4	13.25	3.25	35.6	3.66	23.54	3.54

figure 1-c1

Control	41.2	5.23
Epileptic	45.6	5.36
Jujubosite A	42.6	3.69

figure 1-c2

Control	12.5	2.12
Epileptic	42.2	3.56
Jujubosite A	28.6	3.89

figure 1-d1

Control	35.6	5.23
Epileptic	38.9	3.68
Jujubosite A	32.8	5.55

figure 1-d2

Control	305.6	15.26
Epileptic	100.56	18.6
Jujubosite A	185.9	16.8

figure 2-a

Control	1	0.085
Epileptic	3.23	0.24
Jujubosite A	1.75	0.33

figure 2-b

Control	1.54	0.085
Epileptic	2.95	0.08
Jujubosite A	2.06	0.12

figure 2-c

Control	15.65	2.52
Epileptic	75.65	6.78
Jujubosite A	53.24	5.47

figure 2-d

Control	321.21	32.2
Epileptic	98.56	10.56
Jujubosite A	186.6	20.5

figure 2-e

Control	95.65	6.58
Epileptic	19.85	5.64
Jujubosite A	46.53	8.9

figure 2-f

Control	86.2	3.56
Epileptic	12.36	5.64
Jujubosite A	48.6	7.08

figure 3-a

Control	16.5	3.68
Epileptic	86.5	8.96
Jujubosite A	54.68	8.75

figure 3-b

Control	21.5	4.21
Epileptic	78.6	5.86
Jujubosite A	58.6	6.78

figure 3-c

Control	18.9	3.98
Epileptic	135.5	11
Jujubosite A	86.7	8.9

figure 3-d

Control	25.6	2.5
Epileptic	75.6	5.6
Jujubosite A	53.6	2.45

figure 4-1

Control	1	0.12
Epileptic	4.65	0.24
Jujubosite A	2.42	0.14

figure 4-2

Control	1	0.052
Epileptic	3.16	0.12
Jujubosite A	1.65	0.08

figure S2a-1

Control	1	0.06
Jujubosite A	1.05	0.12

figure S2a-2

Control	1.54	0.12
Jujubosite A	1.478	0.15

figure S2a-3

Control	16.45	3.45
Jujubosite A	18.65	3.56

figure S2a-4

Control	321.21	32.2
Jujubosite A	300.8	28.6

figure S2a-5

Control	93.5	10.5
Jujubosite A	100.5	8.9

figure S2a-6

Control	83.2	5.6
Jujubosite A	90.2	6.5

figure S2b-1

Control	15.89	2.56
Jujubosite A	16.48	1.56

figure S2b-2

Control	18.6	3.5
Jujubosite A	20.3	2.5

figure S2b-3

Control	23.5	2.5
Jujubosite A	19.85	3.56

figure S2b-4

Control	20.56	3.5
Jujubosite A	22.65	2.47

figure S3a-1

Control	1	0.065
Epileptic	3.458	0.102
SB203580	2.102	0.124
ravoxertinib	2.214	0.056

figure S3a-2

Control	1.521	0.124
Epileptic	3.145	0.211
SB203580	2.236	0.014
ravoxertinib	2.354	0.114

figure S3a-3

Control	19.541	1.254
Epileptic	62.341	3.657
SB203580	59.472	1.567
ravoxertinib	60.214	4.214

figure S3b-1

Control	300.547	10.214
Epileptic	100.651	5.678
SB203580	198.546	7.08
ravoxertinib	210.564	10.567

figure S3b-2

Control	98.685	20.541
Epileptic	23.587	3.987
SB203580	62.354	7.08
ravoxertinib	66.357	3.578

figure S3b-3

Control	79.6	5.6
Epileptic	10.56	3.2
SB203580	35.9	7.08
ravoxertinib	40.6	3.5

figure S3c-1

Control	20.567	2.541
Epileptic	87.658	5.641
SB203580	62.314	2.316
ravoxertinib	65.386	1.235

figure S3c-2

Control	21.3	1.25
Epileptic	82.534	10.524
SB203580	58.964	6.784
ravoxertinib	62.347	7.854

figure S3c-3

Control	24.521	3.654
Epileptic	148.678	5.687
SB203580	73.689	6.147
ravoxertinib	78.612	6.654

figure S3c-4

Control	22.5	3.5
Epileptic	79.6	6.5
SB203580	59.6	3.5
ravoxertinib	60.3	3.5
