# Supplementary material for: Jujuboside A Exhibits an Antiepileptogenic Effect in the Rat Model via Protection against Traumatic Epilepsy-Induced Oxidative Stress and Inflammatory Responses
Source: Evid Based Complement Alternat Med. 2022 Sep 9;2022:7792791. doi: 10.1155/2022/7792791 (PMC9481365; doi:10.1155/2022/7792791)
Supplement: Supplementary Materials — We provided Figures S1–S4 and Table S1 in the supplementary material for comprehensive analysis. Part of the raw data (analyzed by OriginPro) has also been provided in the. [file 7792791.f1.zip › 7792791.f1/Supplementary material (1).docx]

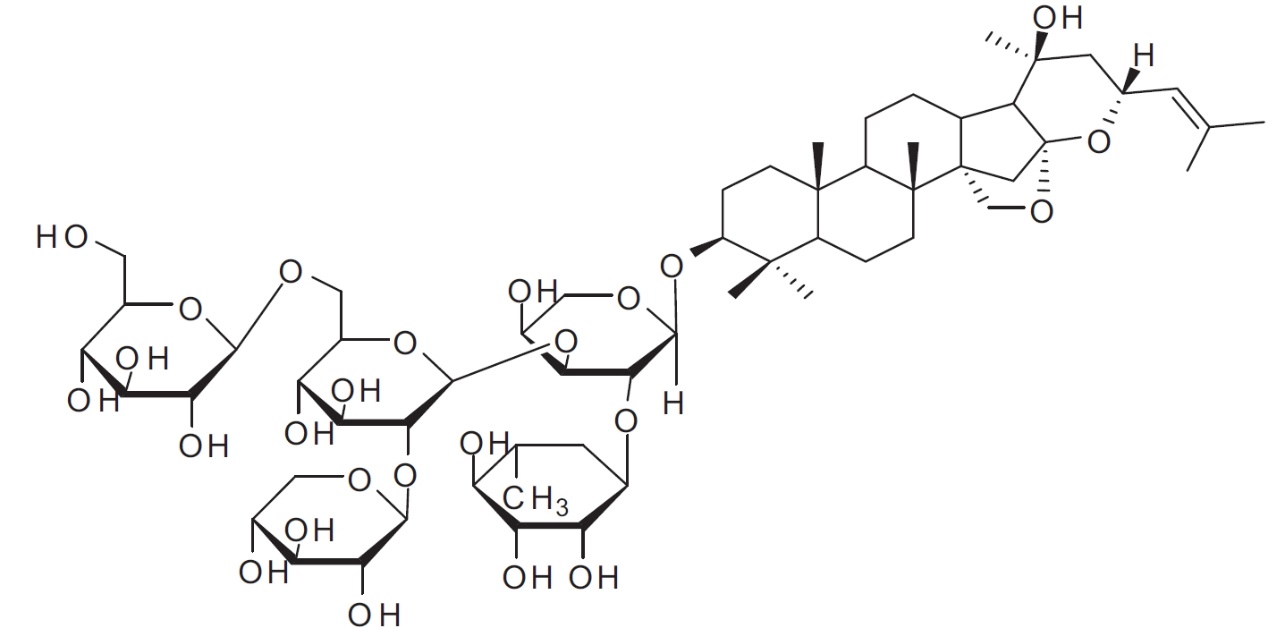


**Figure S1**. The chemical structure of Jujuboside A.


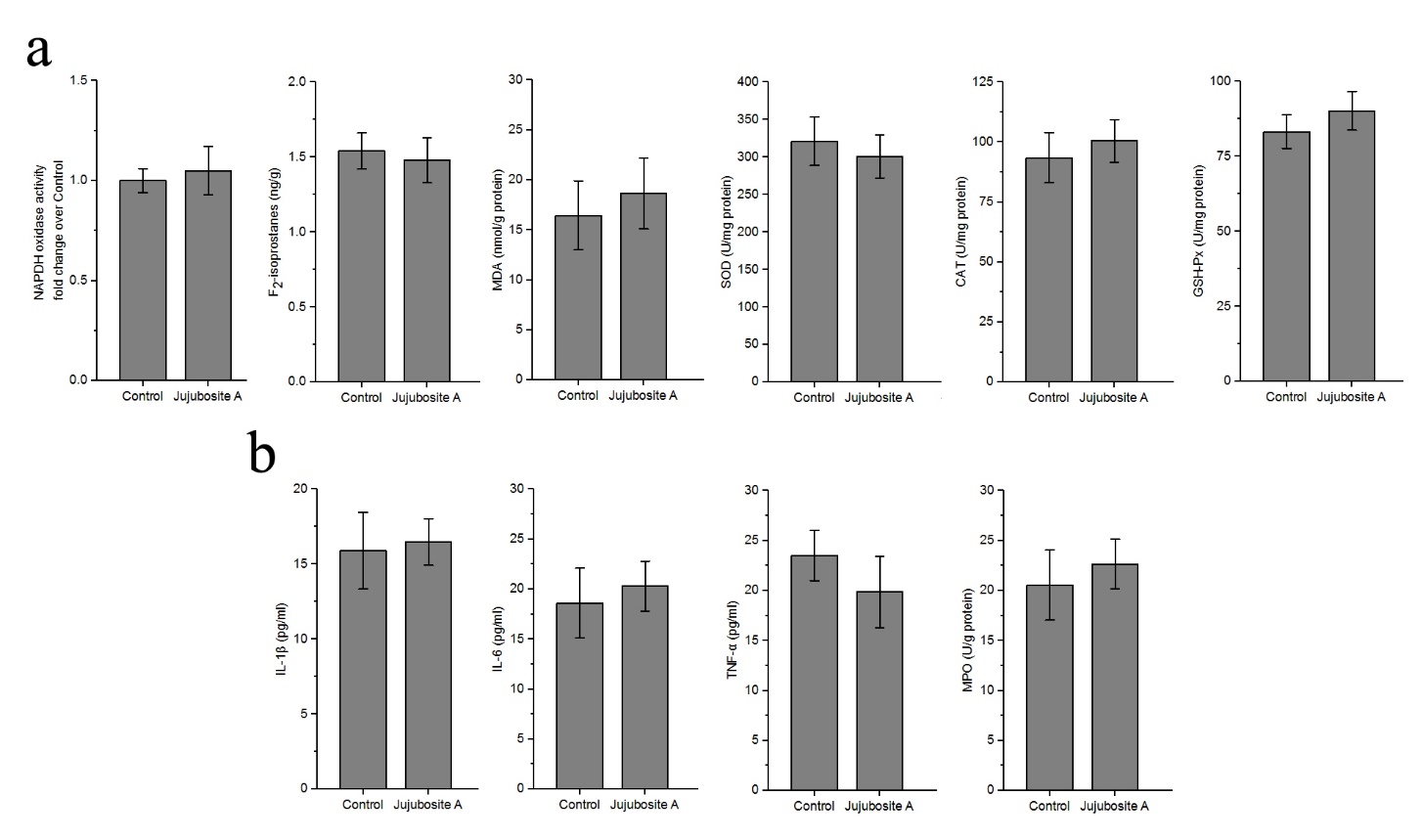


**Figure S2.** Jujuboside A has no effects on healthy rats. (a) Effects of Jujuboside A on local oxidative stress in healthy rats. (b) Effects of Jujuboside A on inflammatory response in healthy rats. 𝑛=3 per group.


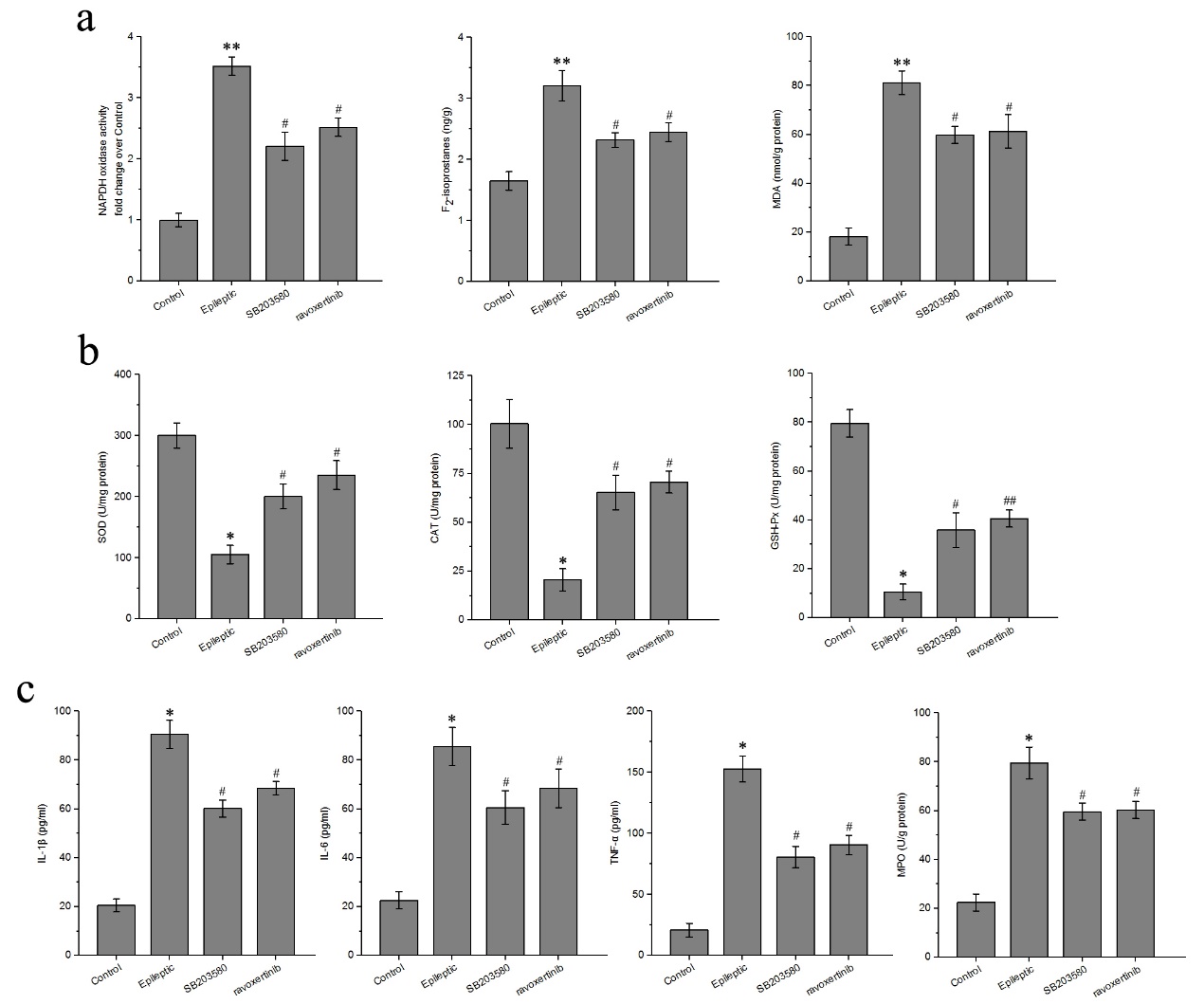


**Figure S3. Effects of inhibitors of MAPK pathways on FeCl_3_-induced epileptogenesis.** (a) Inhibitors of MAPK pathways reduced NADPH oxidase activity and the levels of F_2_-isoprostanes and MDA in mice following FeCl_3_-induced epileptogenesis. (b) Inhibitors of MAPK pathways enhanced the activity of SOD, CAT and GSH-Px in mice following FeCl_3_-induced epileptogenesis. (c) Inhibitors of MAPK pathways decreased the levels of IL-1β, IL-6, TNF-α and MPO in mice following FeCl_3_-induced epileptogenesis. ^*^*P*<0.05 and ^**^*P*<0.01 compared with the Control group; ^#^*P*<0.05 and ^##^*P*<0.01 compared with the Epileptic group. 𝑛=3 per group.

**
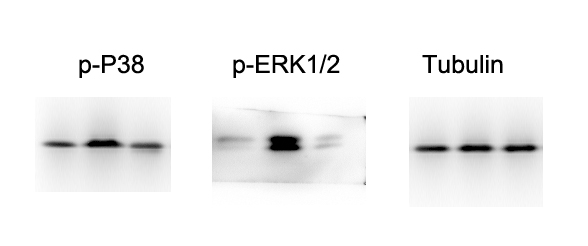
**

**Figure S4. The uncropped Western blot scans.**

**Table S1** Criteria used for classifying different grades of seizures manifested by epileptic and Jujuboside A-treated experimental rats.

|  | Control | Grade I | Grade II | Grade III | Grade IV |
| --- | --- | --- | --- | --- | --- |
| Electrophysiological observations | free from transient discharges | Isolated spikes | Frequent isolated spikes along with cumulative spikes | Frequent bursts of spikes | Long episodes of  epileptic spikes |
| Behavioral observations | None observed | None observed | Steadfast posture, pause in  behavior | Head nodding movement facial movement | Shivering, head nodding  movements |
| Duration of transient epileptogenic episodes | Nil | >1 s to <5 s | 1-5 s | 1-10 s | 5-20 s |
| Frequency composition/amplitude | Nil | 3 or 4 Hz; <250 μV | 6-7 Hz; <250 μV | 6.5-8 Hz; <250 μV | 7-10 Hz; <250 μV |
